# Risk reduction of diarrhea and respiratory infections following a community health education program - a facility-based case-control study in rural parts of Kenya

**DOI:** 10.1186/s12889-020-08728-z

**Published:** 2020-04-29

**Authors:** Miriam Karinja, Raymond Schlienger, Goonaseelan Colin Pillai, Tonya Esterhuizen, Evance Onyango, Anthony Gitau, Bernhards Ogutu

**Affiliations:** 1 Swiss Tropical and Public Health Institute (Swiss TPH), Basel, Switzerland; 2 University of Basel, Basel, Switzerland; 3 Center for Research in Therapeutic Sciences (CREATES), Strathmore University, Nairobi, Kenya; 4 Quantitative Safety and Epidemiology, Chief Medical Office & Patient Safety, Novartis Pharma AG, Basel, Switzerland; 5 CP+ Associates GmbH, Basel, Switzerland; 6 Division of Clinical Pharmacology, Department of Medicine, University of Cape Town, Cape Town, South Africa; 7 Division of Epidemiology and Biostatistics, Faculty of Medicine and Health Sciences, Stellenbosch University, Cape Town, South Africa; 8 The Children’s Investment Fund Foundation, Nairobi, Kenya; 9 Centre for Clinical Research, Kenya Medical Research Institute, Nairobi, Kenya

**Keywords:** Diarrhea, Respiratory infection, Health education, Hygiene, Case-control study

## Abstract

**Background:**

Diarrheal and acute respiratory infections remain a major cause of death in developing countries especially among children below 5 years of age. About 80% of all hospital attendances in Kenya can be attributed to preventable diseases and at least 50% of these preventable diseases are linked to poor sanitation. The purpose of this study was to assess the impact of a community-based health education program, called Familia Nawiri, in reducing the risk of diarrhea and respiratory infections among people living in three rural Kenyan communities.

**Methods:**

Cases were defined as patients attending the health facility due to diarrhea or a respiratory infection while controls were patients attending the same health facility for a non-communicable disease defined as an event other than diarrhea, respiratory infection. Adjusted odds ratios (ORs) with 95% confidence intervals (CIs) were calculated using a logistic regression model to assess the risk of diarrheal or respiratory infection in association with exposure to the health education program.

**Results:**

There were 324 cases and 308 controls recruited for the study with 57% of the cases and 59% of the controls being male. Overall, 13% of cases vs. 20% of control patients were exposed to the education program. Participants exposed to the program had 38% lower odds of diarrhea and respiratory infections compared to those not exposed to the program (adjusted OR 0.62, 95% CI 0.41–0.96). A similar risk reduction was observed for participants in the study who resided in areas with water improvement initiatives (adjusted OR 0.65, 95% CI 0.47–0.90). Variables in the adjusted model included water improvement projects in the area and toilet facilities.

**Conclusion:**

Findings from this study suggest participants exposed to the education program and those residing in areas with water improvement initiatives have a reduced risk of having diarrhea or respiratory infection.

## Background

Diarrhea and acute respiratory infections remain a major cause of death in low income countries especially among children below 5 years of age accounting for about 9 and 13% of annual deaths respectively [1–3]. In Kenya, about 80% of all hospital attendances can be attributed to preventable diseases. Among these preventable diseases, approximately 50% of them are water, sanitation and hygiene related. Diarrheal diseases are ranked among the top ten causes of morbidity and mortality in Kenya and in most rural healthcare facilities; diarrhea is ranked third among the leading causes of outpatient attendance [4]. Furthermore, in Kenya diarrheal diseases cause 16% of deaths among children below 5 years of age followed by pneumonia [4].

While notable progress has been made - including a 53% decrease in the worldwide mortality rate of children below 5 years between 1990 and 2015 [5] - still about 1 in 12 children die before their 5th birthday in low income countries compared to 1 in 147 in the developed world [6]. The Global Burden of Disease 2015 policy report also highlighted “Exposure to poor sanitation, indoor air pollution, and childhood under nutrition has dropped, resulting in dramatic declines in the burden of diarrhea and pneumonia in children” [7]. At a country level however, this should not result in any decrease in their infection control efforts, and there remains a need to identify interventions against common diseases affecting children such as diarrhea and respiratory infections [8]. The negative impact of poor sanitary conditions on health remains as much of a public health concern in the developing world today as when it was a surprising revelation to London during John Snow’s pioneering epidemiological work on cholera infection over 150 years ago [9].

Hand washing is one of the best studied hygiene practices in resource constrained settings. Findings from randomized controlled trials (RCTs) and observational studies on hand washing with soap have shown reduction in diarrhea of between 30 and 47% [10, 11]. A 2006 quantitative systematic review of seven homogenous interventional studies reported a 16% (95% confidence interval [CI] 11–21%) reduced risk of respiratory infections through hand washing with soap [12]. Similar findings of a 47% risk reduction of diarrhea were also reported in a 2003 systematic review on the effect of hand washing with soap [13] and a 35% reduction in incidence of diarrhea in a 2017 RCT on hand washing with soap and Water, Sanitation and Hygiene (WASH) education intervention [14]. Recent RCTs assessing interventions promoting healthy behaviour related to WASH in improving child health outcomes like diarrhea have yielded negative results [15–17]. One of the studies found no benefit of individual interventions such as hand washing, sanitation, water treatment and nutrition [16] while the other found no additive benefit of the interventions over single interventions [17]. Another RCT conducted in Bihar, India on hand washing with soap among school children and their mothers showed little effect on targeted behaviour such as hand washing with soap after defecation and using soap for bathing [18].

A World Bank review found hygiene promotion including hand washing to be the most cost effective intervention for disease prevention at a cost of approximately $3.4 for each disability-adjusted life-year saved [19]. Despite the large body of evidence showing the cost effectiveness and benefits of hygiene promotion in reducing the burden of infectious diseases, there remains low investments in hygiene in the public health, water and sanitation sector [10]. Even more puzzling is the fact that despite the evidence on the ability to prevent diseases, hand washing with soap is still not common practice with some studies reporting 5–15% usage even at the critical times such as after toilet use [20]. This highlights that knowledge alone is insufficient when it comes to changing behaviour and also acknowledges that changing deep seated, private and culturally embedded hygiene practices is a complex and uncertain process [21].

This study evaluates a community-based health education program implemented in selected parts of Kenya. The program was implemented by Familia Nawiri (a Swahili term for “healthy family”), a social venture program initiated in Kenya by Novartis, a multinational pharmaceutical company in three rural settings in Embu, Kirinyaga and Nakuru counties [22]. The education sessions were based on the assumption that through continuous education of community groups, the program would be able to conjure positive change in health related behaviour and thus, reduce the risk of preventable diseases such as diarrheal and respiratory diseases. There are various theories that have been developed and used to explain the relationship of factors that affect health related behaviour. These theoretical behaviour models have been applied to health education and health promotion - among other areas - sometimes with considerable success [23]. Though not applied to the Familia Nawiri program implementation, the RANAS (Risk, Attitudes, Norms, Abilities, Self-Regulation) model for behaviour change which is based on several behaviour change theories [24] has been applied to change behaviour in WASH with good success [25, 26]. The model groups factors that need to be favorable in order for a new behaviour to emerge into five categories (risk factor, attitude factors, norm factors, ability factors and self-regulation factors) and these are matched with specific behaviour change interventions [24, 27].

The purpose of this study was to evaluate the Familia Nawiri community based health education program in reducing the risk of diarhoea and respiratory infections by comparing the odds of exposure to the education program in cases diagnosed with diarhoea or respiratory infections to the odds of exposure to the education program in controls without diarhoea or respiratory infections.

## Methods

### Program implementation

The program focused on community-based health education at a group level as a way of encouraging lasting behaviour change. The groups comprised of women, men, church, youth and table banking (informal money saving) groups with majority being women. The choice to deliver the health education through the group platform was based on the high prevalence of self-help and informal money saving group among others in the rural setting [28, 29]. Since the groups already existed prior to implementation of the program, this provided an easier entry route into the community with larger audience as opposed to individual house visitations. Existing groups in the project sites were mapped and approached for permission to deliver health education sessions during their usual meeting times. The education sessions were delivered by trained health educators who resided in the same communities as the attendees at the education sessions. The health educators had as a minimum a secondary school education level. Prior to initiation of the program, the health educators received training focused on content of hygiene education topics, communication skills and styles, adult learning and facilitation skills, time management and the overall format and structure of a group education session as they would occur in the community. After the training, the health educators were deployed to their communities where they provided health education to the various existing organized groups in their communities.

The hygiene education curriculum comprised two parts, namely, personal and environmental hygiene. Key messages under personal hygiene included body hygiene, dental hygiene, proper hand washing practices and the importance of good hygiene in preventing contagious diseases. Messages on hand washing were coupled with a demonstration on proper hand washing procedure. Key messages on environmental hygiene included main water sources, water treatment methods, importance of clean environment including household surfaces, floors, clothes, outside living area, bathroom, latrines, cooking areas and dishes. There was also an emphasis on having improvised hand washing stations with soap near the toilet facilities to encourage hand washing at critical times, including a demonstration on how to construct these hand washing stations. Each group received at least 2 sessions on each part of the hygiene curriculum, each session lasting 20–40 min. The education sessions took place during the regular meetings for the different groups. The sizes of the groups differed depending on the purpose of the group’s existence but mainly ranged from 10 to 200 - though not all members attended the education sessions. For this study we are not able to report the actual number of people who attended the awareness sessions as these were not recorded at the program level. The education sessions were conducted in a participatory manner with both the health educators and the participants contributing to the questions and discussions.

### Study design and setting

A health facility-based case-control design was used to assess the impact of the education program on reducing the risk of diarrhea and respiratory infections. The rationale for this approach was based on the fact that there were no baseline outcome measurements available to allow for a before and after design. An RCT or a prospective observational study would be a more appropriate design in certain situations to evaluate the effect of an intervention. However, in our case, the program sponsors designed and implemented the program without a specific plan to do an assessment of the effect of the program. When we decided to assess the program, it had already been implemented, and therefore, any prospective assessment was not feasible anymore, and therefore we decided to conduct a case-control analysis bearing in mind the limitations inherent to that design.

The study was carried out in three counties in Kenya, namely Nakuru (Molo constituency), Kirinyaga (Mwea constituency) and Embu (Manyatta constituency) which were the initial pilot sites for the Familia Nawiri community health education program.

### Selection of cases and controls

A total of six health facilities in Nakuru County, eight in Kirinyaga County and five in Embu County were selected based on their location in the target areas where the education program had been implemented. Participants were eligible for the study if they attended the health facility during the study period (June 2014 to November 2014) and if they were residents of the same village for more than 6 months. Children who were accompanied to the health facilities by a parent or a legal guardian who was willing to take part in the study and able to provide written informed consent on behalf of the minor were also included in the study. Where children were recruited into the study, data on the main caregiver such as attendance of Familia Nawiri Health Education program were obtained from the accompanying parent or guardian. Cases were defined as patients attending the health facilities because of diarrhea or respiratory infection. In line with the definition by the Ministry of Health of Kenya [30], the world health organization (WHO) definition for diarrhea namely, having three or more loose or liquid stools per day or more frequently than normal for the individual was applied [31]. Respiratory infections were defined as patients having one or more of the following diagnoses and/or symptoms: Pneumonia, bronchitis, and cold/cough plus any of the following other symptoms: difficulty breathing, chest pain, sore throat, sneezing, or runny nose [32]. Controls were defined as patients attending the same health facility within 2 days of a case for a ‘non-communicable disease’ reason, i.e. an event other than diarrhea, respiratory infection.

### Data collection

Mobile electronic data collection was employed for this study. The questionnaire used was adapted from a previously published World Health Organization questionnaire [33] that had been used to evaluate a water, sanitation and hygiene education intervention. The questionnaire was programmed in the Mezzanine mhealth software platform [34] and deployed onto Android mobile phones. After providing a written informed consent, the participants or their parents or guardians were asked questions from the electronic questionnaire and all the answers were captured on the mobile devices. The completed questionnaire was then automatically uploaded to a host server. In places where there was no network coverage, the completed questionnaires were stored securely on the device and later automatically uploaded when a signal was found. The data collectors were community health extension workers (CHEWs) who administered the questionnaire to the participants at the health facility. The case and control patients were referred to the CHEWs for participation in the study by the clinicians at the facility after consultation and diagnosis.

Data collected included socio-demographic factors, hygiene practices, and behaviour (e.g. age, sex, education level, number of people living in the house, type of house roof, floor and walls, distance to health facility, mode of transport to health facilities, main sources of water, storage of drinking water and water treatment practices). Personal hygiene was assessed using a series of practice and behaviour questions. Seven of the questions related to critical hand washing time points such as before eating, after handling child’s stool and after visiting the toilet. The behaviour was then evaluated as a whole and awarded a composite score with each correct answer yielding 1 point while every wrong answer yielding zero points. Factors causing diarrhea and the health seeking behaviour for illness due to diarrhea and respiratory infections were assessed in a similar fashion.

The CHEWs underwent an intensive one-day training prior to commencing data collection. The training focused on the data collection protocol including basics of conducting interviews, pretesting of the survey instruments for suitability and appropriateness, field logistics, general orientation to using the mobile devices and the data collection software, accessing the questionnaire on the phone, trouble shooting and handling technical difficulties with the mobile device. Special attention was paid during training on interview techniques especially the avoidance of asking ‘leading’ questions. A pilot test in which the CHEWs conducted interviews with each other and inputting dummy data into the app, was conducted prior to starting data collection in order to allow them to gain familiarity with the app, to test our processes and to allow minor adjustments to the data collection instrument.

### Sample size estimation

The sample size for this frequency-matched case-control study was estimated using formulary as described by [35] for a binary outcome analysis using logistic regression. We calculated that a minimum sample size of 272 for either a case or control will be required based on 2-sided testing with a 0.05 level of significance and 80% power in order to detect a risk reduction of 0.5 (OR = 0.5). The assumption made in calculating the sample size was that 20% of the controls would have been exposed to the Familia Nawiri hygiene education program. Accounting for 5% non-response rate, the final sample size was 286 for cases and 286 for controls; a minimum sample size total of 572 participants were therefore needed. Enrolment of participants from each health facility was not done in proportion to the population or numbers attending the health facility, instead we divided the overall sample size equally across the three sites where the study was conducted. In order to minimize selection bias and ensure controls were of a similar source population as the cases, participants were frequency-matched on the basis of recruitment by health facility and the time of diagnosis with controls being recruited within 2 days of a case. For this study we did not match the cases and the controls based on factors such as age and gender to avoid over matching and also difficulty in recruiting participants. However, we adjusted for these factors during analysis stage.

### Data analysis

Data were entered into Stata software, StataCorp.2013 [36] (for analysis. The age of the participants and the age of the mother were reported using median and interquartile range (IQR), while all the other study variables which were categorical were reported as percentages (%). Pearson’s Chi-squared test was used for comparing categorical variables. Logistic regression analysis was used with diarrhea or respiratory infection as the dependent variable and attending Familia Nawiri health education sessions as the independent variable to estimate crude odds ratios (ORs) with corresponding 95% CIs in cases compared to controls. The participant needed to have attended at least one session of Familia Nawiri Health education intervention to be considered exposed. Other predictor variables included in the initial model based on scientific literature were: age, gender, having toilet facility, having other water improvement initiatives in the same localities (this included initiatives through government, community or private sector such as providing piped water into the homesteads or community tapes, sinking of boreholes and water treatment products), having attended any other health education initiative in the same locality, and distance to health facility. For categorical variables, missing responses were put in a separate category and included in the regression model. There were no missing values for the continuous variables. To adjust for confounding and/or effect modification a backward stepwise deletion approach was used whereby variables were dropped one by one starting with those with highest *p*-value until a final model was obtained.

## Results

Overall, 640 questionnaires were completed by 330 cases and 310 controls. Out of the completed questionnaires, 8 (1.3%) were excluded on the basis of incomplete interviews, leaving 632 for the final analysis (324 cases and 308 controls). At least 153 (47%) of the cases and 143 (46%) of the controls from Embu County, while 85 (26%) of the cases and 82 (27%) of the controls were from Kirinyaga and 86 (27%) cases and 83 (27%) of the controls were from Nakuru County. The median age of cases was 20 years (IQR: 2–35 years) while that of controls was 23 years (IQR: 2–35 years). The proportion of males was higher, both in cases (57%), and controls (59%). Among the cases, 79% presented with respiratory tract infections while 21% presented with diarrhea. Among the controls, the most frequently mentioned presenting conditions included accident or injury 11%, Family planning services 11%, skin conditions 11%, burns 5%, sexually transmitted infections 5%, hypertension 4%, fever 4%, dental problems 2%, diabetes 1% among others. Table 1 presents an overview of the characteristics of the case and control patients. The majority of the participants had up to primary school as their highest level of education (cases 49 and 52% of the controls). Among the cases, a lower proportion, compared to controls, had attended Familia Nawiri education sessions (13% vs. 20%, respectively). We assessed for association between the number of sessions attended and found no statistically significant difference between cases and controls who attended just one session or more than one session. About 34% of the cases compared to 45% of the controls reported residing in areas where there were water improvement initiatives. There was no difference noted in knowledge of causes of diarrhea, recognition of danger signs or critical times to seek medical attention for diarrhea and respiratory infection between the cases and the controls. The results from the logistic regression model indicate that exposure to Familia Nawiri health education reduced the risk of being a case with diarrhea or respiratory infection (adjusted OR 0.62, 95% CI: 0.41–0.96 (*p* = 0.03) [adjusted for other water improvement projects and presence of toilet facility]. Table 1 provides characteristics of cases and controls and crude ORs. Table 2 shows adjusted ORs of predictor variables used to arrive at the final statistical model.

**Table 1:** Characteristics of cases and controls and crude odds ratio for diarrhea and respiratory infections in cases and controls exposed to Familia Nawiri Health Education Program

Variable	Cases (*n* = 324)	Controls (*n* = 308)	Crude	
			OR (95% CI)	***P*** value
Median age of participant (IQR)	20 (2–35)	23 (2–35)	1.00 (0.99–1.01)	0.75
Gender, n (%)				
Male	186 (57.41)	183 (59.42)	1.00 (reference)	
Female	138 (43.59)	125 (40.58)	1.09 (0.69–1.37)	0.89
Highest level of education, n (%)				
Kindergarten and Primary level	158 (48.77)	161 (52.27)	1.00 (reference)	
Post-primary/vocational	6 (1.85)	5 (1.62)	1.60 (0.43–5.90)	0.48
Secondary/‘a’ level	120 (37.04)	111 (36.04)	1.11 (0.78–1.58)	0.57
College and University	27 (8.33)	19 (6.17)	1.77 (0.91–3.48)	0.09
Missing	13 (4.01)	12 (3.90)		
Water improvement interventions, n (%)				
No	204 (62.96)	163 (52.92)	1.00 (reference)	
Yes	111 (34.26)	137 (44.48)	0.68 (0.48–0.95)	0.03
Missing	9 (2.78)	8 (2.60)		
Distance to health facility, n (%)				
10 mins	41 (12.65)	38 (12.34)	1 (reference)	
30 min	196 (60.49)	205 (66.56)	0.78 (0.47–1.29)	0.33
> 1 h	84 (25.93)	64 (20.78))	1.06 (0.59–1.92)	0.83
Missing	3 (0.93)	1 (0.32)		
Exposure to Familia Nawiri, n (%)				
No	272 (83.95)	239 (77.60)	1.00 (reference)	
Yes	43 (13.27)	61 (19.81)	0.62 (0.40–0.96)	0.03
Missing	9 (2.78)	8 (2.60)		
Toilet facility, n (%)				
No	8 (2.47)	2 (0.65)	1.00 (reference)	
Yes	308 (95.06)	301 (97.73)	0.23 (0.05–1.11)	0.07
Missing	8 (2.47)	5 (1.62)		
Improved water sources, n (%)				
No	66 (20.37)	62 (20.13)	1.00 (reference)	
Yes	251 (77.47)	242 (78.57)	0.94 (0.62–1.42)	0.76
Missing	7 (2.16)	4 (1.30)		
Attended any other hygiene education sessions apart from Familia Nawiri				
No	211 (65.12)	215 (69.81)	1.00 (reference)	
Yes	104 (32.10)	85 (27.60)	1.08 (0.57–1.13)	0.69
Missing	9 (2.78)	8 (2.60)

**Table 2:** Final model showing crude and adjusted odds ratio for diarrhea and respiratory infections in cases and controls exposed to Familia Nawiri Health Education Program

Variable	Cases (*n* = 324)	Controls (*n* = 308)	Crude		Adjusted^a^	
			OR (95% CI)	***P*** value	OR (95% CI)	***P*** value
Exposure to Familia Nawiri, n (%)						
No	272 (83.95)	239 (77.60)	1.00 (reference)		1.00 (reference)	
Yes	43 (13.27)	61 (19.81)	0.62 (0.40–0.96)	0.03	0.62 (0.41–0.96)	0.03
Median age (IQR)	20 (2–35)	23 (2–35)	1.00 (0.99–1.01)	0.75	1.00 (0.99–1.00)	0.79
Gender, n (%)						
Male	186 (57.41)	183 (59.42)	1.00 (reference)		1.00 (reference)	
Female	138 (43.59)	125 (40.58)	1.09 (0.69–1.37)	0.89	0.98 (0.71–1.37)	0.93
Water improvement interventions, n (%)						
No	204 (62.96)	163 (52.92)	1.00 (reference)		1.00 (reference)	
Yes	111 (34.26)	137 (44.48)	0.68 (0.48–0.95)	0.03	0.65 (0.47–0.91)	0.01

^a^Adjusted for all variables in the table

## Discussion

This study set out to evaluate the Familia Nawiri community health education program on risk reduction for diarrhea and respiratory infections. Findings from the study revealed favorable results for this hygiene education program. Overall, after adjusting for potential confounders, participants exposed to the hygiene education program had 38% lower odds of having diarrhea or a respiratory infection compared to participants who were not exposed to the hygiene education program (adjusted odds ratio 0.62, 95% CI: 0.41–0.96). Our findings are consistent with estimates from other studies assessing effects of hand washing with soap and WASH educational intervention on reducing the incidence of diarrhea and respiratory infections [11, 13, 14]. On the other hand, recent RCTs assessing interventions promoting healthy behaviour related to WASH, in improving child health outcomes like diarrhea and proper hand washing behaviour have yielded negative results [15–18]. For instance, a trial conducted in Rwanda assessing the impact of community-led health clubs promoting WASH interventions reported no effect on care-giver reported diarrhea among children below 5 years across the three arms of the study (control group, eight community health club sessions group and 20 community health club sessions group) [15]. A trial conducted in Kenya with seven arms (including: control arm, water intervention, sanitation intervention, hand washing intervention, combination of water, sanitation and hand washing intervention, nutrition intervention and combination of all the interventions [water, sanitation, hand washing and nutrition]) found no reduction in diarrhea in any of the intervention arms [16]. However, a trial from Bangladesh – with similar intervention arms as the study in Kenya – reported reduction in diarrhea in all intervention arms, except the intervention arm receiving water treatment only [17]. Although some of these recent studies have reported no effect of the interventions tested, the benefits of WASH for diarrheal diseases and other health outcomes should not be underestimated [37, 38]. The findings may also not be generalizable across all contexts and therefore should be viewed in light of the specific interventions and setting [37, 39, 40]. With regards to the intervention and the approach used to deliver the intervention, comparable examples in other low and middle income countries in Africa, have been provided by Sinharoy et al., Waterkeyn and Cairncross, and Lewycka et al. [15, 41, 42]. However, findings from these interventions are varied. Cairncross, and Lewycka et al. [41, 42] found that community health clubs and women’s group interventions can be effective in achieving high levels of health knowledge and hygiene behaviour change in Zimbabwe and also to improve maternal and child health in Malawi. On the other hand, Sinharoy et al., [15] found that the use of community health clubs as implemented under Rwanda’s national Community-Based Environmental Health Promotion Program in western Rwanda had no effect on health outcomes such as diarrhea in children under 5 years old.

It cannot be excluded with certainty that unmeasured bias or confounding e.g. recall bias with respect to exposure to the Familia Nawiri education sessions, residing in areas with water improvement initiatives and attending other health education programs, may have impacted the results in either direction, i.e. either increasing or decreasing the odds ratio. However, since our findings were statistically significant (95% CI: 0.41–0.96, *P* = 0.03), they provide some support of an association between hygiene education and morbidity of diarrhea and respiratory tract infections.

Some of the strengths of this study include the fact that both cases and controls were recruited from the same health facilities, therefore, taking regional practice aspects into consideration. Selection of cases and controls was within 5 days of attending the clinic which helped to minimize potential seasonal influence on the outcomes.

The findings from this case-control study should be interpreted in light of some limitations. There is potential risk to overestimate the health impact as a result of relying on self-reported measurements. This could have been avoided by employing direct observation methods at the household level. However, due to logistical reasons and inconveniences that such a method would entail, we decided to rely on the self-reported measurements. We believe however, that the resulting misinformation would have been non-differential between cases and controls – in other words - any misinformation that occurred while collecting the data at the health facility may have occurred to the same extent for both groups and would therefore rather mask than exaggerate the impact of the intervention. Another limitation for our study is the fact that the intervention was delivered to a highly selective target audience - participants in group activities such as church groups or women’s groups. It can be expected that these people differ in many psychosocial parameters that may not reflect in the measured socio-economic indicators. Misclassification bias with respect to exposure is also very likely for this study. This is mainly because there was no objective way of verifying reported or non-reported exposure to the education sessions. This has a bearing on how the participants recall exposure to the intervention. However, for this study, both the cases and the controls were selected from participants seeking medical attention at health facilities; hence, both cases and controls would have similar concerns regarding the causes of their illness making them comparable and thus minimizing differential recall bias. Approximately 60% of the cases and the controls reported having attended some form of hygiene education session apart from the Familia Nawiri program while 45% of the controls and 34% of the cases reported residing in areas where there had been water improvement initiatives. The presence of these other initiatives may account for the finding of no difference in knowledge of the causes of diarrhea among cases and controls. Although the selected subjects for the study were statistically similar, the fact that most of them were children and young adults could perhaps reflect ability to access a health facility and therefore our findings should be interpreted in that context. Since the cases and controls in this study were selected from health facilities in the area where the health education program had purposefully been implemented, we cannot be certain that the findings from this study apply to people in other geographical locations or other health facilities. It is also important to note that recruitment at the health facility level probably shifts to more severe cases that require attendance of the health facility. Therefore, it is not clear whether the results would also apply to less severe cases of diarrhea and respiratory infections. Another limitation for this study is the fact that we did not consider the difference in population size of the three regions when enrolling participants to the study which could in turn affect the validity of the results. A final limitation for this study is the fact that we did not utilize theory of change in assessing the effect of the community health education program as the assessment was largely focused on clinical health outcomes and nor was an implementation framework utilized in the implementation of the community health education program. We are therefore limited in our understanding of how or why changes happened as a result of the implemented community health education program or which aspects of the program, if not all were effective.

## Conclusion

This study was a first attempt to assess the effect of the Familia Nawiri community health education program. We found that being exposed to the education sessions and residing in areas with water improvement initiatives were both associated with lower odds of attending a health facility due to diarrhea or a respiratory infection. The findings have implications for planning and implementing community based health education, water, and sanitation and hygiene interventions as they indicate the need for information, education and communication (IEC) activities tailored to the social and cultural context and infrastructure development.

## Data Availability

The datasets used and/or analyzed during the current study are available from the corresponding author on reasonable request.

## Abbreviations

OR: Odds Ratio
CI: Confidence Interval
WHO: World health Organization
RCT: Randomized Controlled Trial
WASH: Water Sanitation and Hygiene
CHEWs: Community Health Extension Workers
IQR: Interquartile range
AMREF: Africa Medical and Research Foundation
IEC: Information, Education and Communication
